# Automated artificial intelligence-enabled proactive preparedness real-time system for accurate prediction of COVID-19 infections— Performance evaluation

**DOI:** 10.3389/fmed.2022.871885

**Published:** 2022-08-30

**Authors:** Leila Ismail, Huned Materwala, Yousef Al Hammadi, Farshad Firouzi, Gulfaraz Khan, Saaidal Razalli Bin Azzuhri

**Affiliations:** ^1^Intelligent Distributed Computing and Systems (INDUCE) Laboratory, College of Information Technology, United Arab Emirates University, Al Ain, United Arab Emirates; ^2^Department of Computer Science and Software Engineering, College of Information Technology, United Arab Emirates University, Al Ain, United Arab Emirates; ^3^National Water and Energy Center, United Arab Emirates University, Al Ain, United Arab Emirates; ^4^Department of Information System and Security, College of Information Technology, United Arab Emirates University, Al Ain, United Arab Emirates; ^5^Department of Electrical and Computer Engineering, Duke University, Durham, NC, United States; ^6^Department Medical Microbiology and Immunology, College of Medicine and Health Sciences, Tawam Hospital, Al Ain, United Arab Emirates; ^7^Department of Computer System and Technology, Faculty of Computer Science and Information Technology, University of Malaya, Kuala Lumpur, Malaysia

**Keywords:** automated artificial intelligence (Auto-AI), coronavirus, COVID-19 infection prediction, deep learning, healthcare, machine learning, performance evaluation, time series

## Abstract

COVID-19 is a contagious disease that has infected over half a billion people worldwide. Due to the rapid spread of the virus, countries are facing challenges to cope with the infection growth. In particular, healthcare organizations face difficulties efficiently provisioning medical staff, equipment, hospital beds, and quarantine centers. Machine and deep learning models have been used to predict infections, but the selection of the model is challenging for a data analyst. This paper proposes an automated Artificial Intelligence-enabled proactive preparedness real-time system that selects a learning model based on the temporal distribution of the evolution of infection. The proposed system integrates a novel methodology in determining the suitable learning model, producing an accurate forecasting algorithm with no human intervention. Numerical experiments and comparative analysis were carried out between our proposed and state-of-the-art approaches. The results show that the proposed system predicts infections with 72.1% less Mean Absolute Percentage Error (MAPE) and 65.2% lower Root Mean Square Error (RMSE) on average than state-of-the-art approaches.

## Introduction

More than 2 years after the outbreak of the COVID-19 disease, the containment of this virus still represents a serious challenge to the world community.^1^ Over half a billion people have been infected worldwide, including more than 6.27 million deaths as of 20 May 2022.^2^ Studies have revealed that COVID-19, caused by severe acute respiratory syndrome coronavirus 2 (SARS-CoV-2), not only affects the lungs of the infected person but also negatively impacts other vital organs such as the brain, heart, liver, pancreas, and kidney (1–3). Effect on the brain can lead to muscular pain and headaches in individuals with a mild infection, whereas in severe cases it could lead to stroke (2). Heart complications due to SARS-CoV-2 include inflammation and dysfunction of muscles and may cause the death of patients suffering from cardiovascular diseases (2). Furthermore, the SARS-CoV-2 virus could lead to pancreatic islet-cell dysfunction (3) causing diabetes (4–6). In addition, it causes liver impairment and acute kidney injury (2). To reduce the spread of the virus, countries have imposed several strict policies and practices, such as travel bans, home confinement, and business closures. These measures showed to be effective in reducing the infection and death rates during this pandemic (7–9). However, too strict measures may lead to income loss, anxiety, and depression on an individual scale, and cause longer-term economic and social hardship on the national scale (10–12). A survey conducted in the United States of America among 5,412 adults showed that 31% of the respondents suffered from anxiety/depression symptoms, 26% from stressor-related disorder symptoms, and 11% considered suicide during the COVID-19 pandemic (13). Strict confinement measures have also shown an adverse effect on students’ mental health. A survey conducted on 69,054 university students during the lockdown in France revealed that 27.5 and 24.7% of the respondents had a high level of anxiety and stress, respectively, 16.1% had severe depression, and 11.4% had suicidal thoughts (14). In addition, individuals often miss routine medical checkups and tests due to confinement, leading to severe health issues, especially in patients suffering from chronic diseases (15). Discontinued daily exercises have been leading to obesity and associated health risks (16). Consequently, it becomes crucial to predict infections to gain a better understanding of the growth of the infection curve, and deeper insight into when to enact, relax or terminate these strategies. In addition, infection forecasting allows healthcare organizations to effectively plan the required medical resources enabling smart healthcare (17, 18).

Artificial Intelligence (AI) algorithms have been widely adopted in the medical sector to enable smarter, effective, and efficient healthcare (19). Different AI-based algorithms are used for screening, diagnosing, and monitoring COVID-19 (20–22) as well as for predicting the number of infections (23–29). Recent studies have used machine/deep learning time series models to predict the spread of COVID-19 infections, based on previous infections, in a few countries. These studies use different prediction models (30). However, considering the difference in the geographical characteristics and social behaviors of the countries under study, we argue that the use of a single prediction model becomes questionable (31). This is because the model is not capable to capture the infection evolution, leading to inaccurate prediction. Such a failure may lead to greater distress and more deaths. Furthermore, these models need to be constantly updated and fail to capture the evolving COVID-19 variants such as omicron.

To address these shortcomings, in this paper, we propose an automated AI-enabled proactive preparedness system for accurate prediction of COVID-19 infection growth in real-time, with no human intervention. The proposed system incorporates an intelligent agent that analyses the temporal distribution of the infection evolution for a city/state/country and maps the prediction model to the corresponding trend using a novel trend-to-model mapping approach. The prediction results by the system aid government and healthcare organizations to be well prepared and proactively tackle the chaotic pandemic situation. For instance, the measures can be relaxed if the prediction shows a decrease in COVID-19 infections, whereas they can be made stricter if an increase in the number of infections is predicted. A detailed real-time infection data acquisition, preprocessing framework, and request-response flow are presented. The performance of the proposed system is compared with state-of-the-art approaches to predict COVID-19 infections in fifteen countries based on the literature.

## Related work

Time series prediction is a useful method that considers the influence of previous infection data to predict future data (31). Different machine learning algorithms have been used to analyze the data of epidemic and pandemic diseases such as influenzas A (H1N1),^3^ B,^4^ measles childhood disease (32), SARS, MERS, and COVID-19 outbreaks, at the country, regional or global level (31). Though any machine learning algorithm can produce reliable results at some level, time series algorithms are the most accurate approaches to studying epidemic and pandemic diseases because of their dynamic and temporal nature (33). Several studies in the literature have proposed the use of different time series machine learning and deep learning algorithms for the prediction of COVID-19 infections in different countries (23–29).

As shown in Table 1, the selection of machine learning algorithms is either not justified (23–26), or based on the popularity of the prediction algorithm (27, 28), or the performance of the algorithm when implemented for some other country (29). However, given the significant difference in the geographical characteristics and social behaviors of the countries, the use of a single algorithm to predict disease spread becomes questionable, as it is highly likely that the algorithm fails to generate accurate predictions (31). Consequently, an algorithm should be selected based on the temporal distribution of the infection evolution data for a country. In this paper, we propose an intelligent agent, integrated within an automated AI system, that will analyze the trend of infection growth in a country, and selects the most accurate learning algorithm. This algorithm predicts COVID-19 infections with the least error for that country than other state-of-the-art algorithms. We compare the performance of our selected algorithm for each country in Table 1 with the outperforming algorithm(s) for that country in the literature.

**TABLE 1:** Summary of COVID-19 infection prediction using time series machine learning and deep learning algorithms.

Work	Considered countries	Considered algorithms	Justification for algorithm selection	Considered period for developing the algorithm	Considered period for validating the algorithm	Outperforming algorithm
Ahmar and Del Val (23)	Spain	ARIMA and SutteARIMA	NR	02/12–04/02 2020	04/03–04/09 2020	SutteARIMA
Gecili et al. (24)	United States and Italy	HLT, ARIMA, TBATS, and cubic smoothing spline		02/22–04/29 2020	02/22–04/29 2020	ARIMA
Shahid et al. (25)	Brazil, Germany, Italy, Spain, United Kingdom, China, India, Israel, Russia, and United States	ARIMA, SVR, LSTM, Bi-LSTM, GRU		01/22–05/10 2020	05/11–06/27 2020	Bi-LSTM
Ayoobi et al. (26)	Australia and Iran	LSTM, Bi-LSTM, Convolutional LSTM, Bi-Convolutional LSTM, GRU, Bi-GRU		(Australia) 01/25–05/20 2020 (Iran) 01/03–06/06 2020	(Australia) 05/21–06/18 2020 (validation) 06/19–08/19 2020 (testing) (Iran) 06/07–07/15 2020 (validation) 07/16–10/06 2020 (testing)	LSTM (Australia) Bi-GRU (Iran)
Ceylan (27)	Italy, Spain, and France	ARIMA	Widely used in literature	02/21–04/15 2020	NA	ARIMA
Singh et al. (28)	Malaysia	ARIMA		01/22–03/31 2020	04/01–04/17 2020	ARIMA
Alzahrani et al. (29)	Saudi Arabia	AR, MA, ARMA, ARIMA	Accurate for other countries	03/02–04/20 2020	NA	ARIMA

AR, AutoRegressive; ARIMA, AutoRegressive Integrated Moving Average; ARMA, AutoRegressive Moving Average; Bi-LSTM, Bidirectional Long Short-Term Memory; GRU, Gated Recurrent Unit; HLT, Holt’s Linear Trend; LSTM, Long Short-Term Memory; MA, Moving Average; NR, Not Reported; NA, Not Applicable; SVR, Support Vector Regression; TBATS, Trigonometric Exponential smoothing state-space model with Box-Cox transformation.

## Materials and methods

### Automated artificial intelligence-enabled proactive preparedness real-time system for accurate COVID-19 infection prediction

**FIGURE 1:**
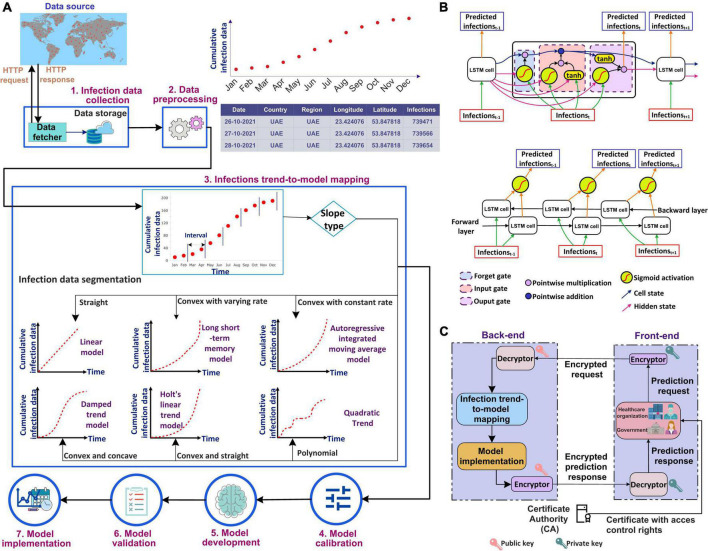
**(A)** Workflow of the proposed automated artificial intelligence-enabled system for infection prediction, **(B)** architecture of long short-term memory (LSTM) cell and Bidirectional-LSTM network used in the proposed system for infection prediction, and **(C)** request-response workflow in the proposed system.

This section presents the workflow of our proposed system for predicting COVID-19 infections along with the steps involved. It explains the method used to select the most accurate model for prediction based on the infection’s trend. The use of a systematic workflow for the problem of infection prediction is the most important for the accurate infection prediction for a given country. Figure 1A shows the seven stages involved in the proposed system. In the following, we explain each stage in detail.

#### Infection data collection

The city-level, state-level, and/or country-level infection data can be collected from a data source that can be either an online repository (such as Johns Hopkins), healthcare organizations, and/or specialized national/international agencies for public health such as World Health Organization (WHO). In this study, we used the Johns Hopkins dataset which includes COVID-19 infections, recoveries, and deaths data from different provinces/states and countries/regions since 22 January 2020. The data fetcher module in our framework sends an HTTP request to a data source for accessing the infection data. A request contains information regarding the city/state/country and the period for which the data is required. In response to the request, the source sends the queried infection data to the fetcher module. The data is fetched at a periodic interval, which can be seconds, minutes, hours, or days depending on the frequency the data is updated in the data source and is stored in a cloud database (34–38).

#### Data preprocessing

The retrieved infection data is preprocessed to make it ready for the machine/deep learning algorithm. This is done by removing irrelevant attributes. As our system predicts the number of infections, the deaths and recoveries data are removed. In addition, preprocessing involves the identification and removal of outliers if any, as well as the identification and handling of missing values. The identification of outliers in infection prediction is important as the learning algorithms are sensitive to outliers and could produce unexpected results (39). The outliers, if present, can be removed using visualization of the infection data plot and/or machine learning approaches based on bagging, boosting, and local outlier factor algorithm (39). The missing values in infection data, if any, can be handled either by removing the corresponding timestamp from the dataset or adding synthetic values. The synthetic values can be generated using statistical methods such as mean, median, and mode, or machine learning approaches such as kNN imputation and rpart (39).

#### Infection trend-to-model mapping

The trend of the preprocessed infection data is analyzed to select the most accurate prediction model that is adaptive to the dynamicity of the evolution of the infection spread. The most accurate model predicts the infections with the least error compared to other models. To analyze the distribution of the infection spread, the infection data is first divided into intervals of equal length as shown in Figure 1A. The slope between each interval is then determined by constructing a linear model between the interval endpoints. If all the data points between the interval endpoints lie below the data points on the linear model, then the slope between the interval endpoints is convex. On the other hand, if all the points between the interval endpoints lie above the points on the linear model, then the slope between the endpoints is concave. The slope is straight if the data points between the interval points lie on the constructed linear model. The distribution of the infection’s trend is then determined based on the slopes, and a corresponding prediction model is selected. ARIMA model is selected to model the infection data following an exponential trend with a constant rate. This is because ARIMA is best suited to capture the exponential behavior of the infection growth (31). For the infection’s data having an exponential trend with varying rates, the Long Short-Term Memory (LSTM) and Bidirectional-LSTM (Bi-LSTM) models are selected as it is capable of capturing the variability in the data (31). The infection data that increase linearly over time are modeled using the Linear Trend (LT) model. For data evolving in a polynomial fashion, the Quadratic Trend (QT) model is selected. HLT model is selected for exponential + linear infection trend. This is because the HLT model is a linear function of trend and slope that captures well the linearity in an exponential trend over time. For the infection’s data with an exponential + damping trend, Damped Trend (DT) model is selected as the damping parameter used by the model provides an accurate prediction of infections for a trend that dampens over time. Figure 1B represents the architectures for the LSTM cell and Bi-LSTM network. The main components of LSTM are the cell state and gates. The cell state transfers the significant previous infection data to the chain of LSTM cells. Gates in LSTM are responsible for storing relevant and removing irrelevant infection data. LSTM consists of three gates: forget, input, and output. All the gates have a sigmoid activation function except the input gate which utilizes a hyperbolic tangent activation function. In LSTM, the forget gate is responsible for removing irrelevant infection data based on the prediction output of the previous cell. The input gate adds the new infection data to the memory cell state. Finally, the output gate generates the output of the cell, i.e., the predicted infections for the next time step based on the current infections and cell state. Bi-LSTM is a recurrent neural network that consists of two LSTM networks, one in the forward direction and another in the backward.

#### Model calibration

The selected prediction model is calibrated for hyperparameter tuning. It is an important stage as non-optimal parameters’ values may increase the resource utilization and execution time for model development and can degrade the model’s convergence and prediction performance.

#### Model development

The dataset is split into training and validation. The most common approach is splitting the dataset into 70 and 30% for training and validation, respectively. The selected algorithm, with the optimal values of the parameters, is then developed using the training dataset.

#### Model validation

The developed model is validated using the validation dataset in terms of Mean Absolute Percentage Error (MAPE) and Root Mean Squared Error (RMSE).

#### Model implementation

The model is implemented in real-time for predicting infections for a city, state, and/or country. The infections trend-to-model mapping, model calibration, and model development are iterative stages. These stages are repeated based on updated and/or new data.

Figure 1C shows the request-response workflow used in the proposed system. The healthcare organizations and the government users interact with the front-end interface of the system. They are authorized based on their Access Control List (ACL) or Role-Based Access Control (RBAC) which is defined by policy. The Certificate Authority (CA) (40–42) generates a pair of public-private keys (43) for all the users. We suggest to use asymmetric cryptosystem such as Elliptic Curve Cryptography (ECC) (44) with the key length of at least 384 bit,^5^ which is equivalent to 7,680 bit RSA (45), for exchanging the key and then 256 bit key of Advanced Encryption Standard (AES), recommended by National Security Agency (NSA), for encryption and decryption ensuring secure communication. In addition, to ensure the integrity of data received from an external source, the SHA3-256 algorithm is used which guarantees that the data has not been modified.

The front-end runs on the user’s premises and communicates with the back-end that consists of our proposed intelligent agent. The prediction request from a user, i.e., the country for which the prediction is required, the prediction period, and the certificate, are sent to the encryptor. The encryptor encrypts the prediction request using the user’s private key. The encrypted request is sent to the intelligent agent in the back-end. The agent decrypts the request using the public key of the request initiator. Once successfully decrypted, the agent analyzes the trend of the infection data for the country and selects the most accurate prediction model. The results of the prediction model are then encrypted by the agent using the initiator’s public key. The encrypted prediction response is sent to the user at the front-end. The response is then decrypted using the user’s private key.

### Implementation of the proposed automated artificial intelligence-enabled system for real-time infection prediction

**FIGURE 2:**
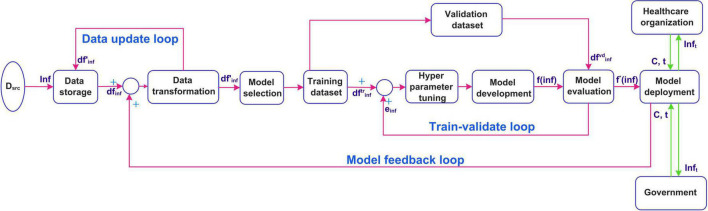
Implementation of the proposed real-time prediction system.

In this section, the implementation of the real-time system is discussed. The suggested implemented diagram is shown in Figure 2. The infection data is collected from different data sources *D*_*src*_ such as the Ministry of Health, Hospitals, and public health agencies (for example WHO). The infection data *Inf* is stored using the data storage component. The raw infection data is stored as a data frame *df*_*inf*_is fed as an input to the data transformation component. The preprocessed data frame d⁢f′i⁢n⁢f is again stored. The transformed data is constantly updated in the storage in real-time using a data update feedback loop. The preprocessed data is then divided into training $d\,f_{i\,n\,f}^{t\,r}$ and validation $d\,f_{i\,n\,f}^{v\,d}$ datasets. A model is selected by the intelligent agent based on the temporal distribution of the infection data evolution. The selected model *f*(*inf*)is developed using$d\,f_{i\,n\,f}^{t\,r}$. The performance of the model is evaluated using$d\,f_{i\,n\,f}^{v\,d}$. The model development is a feedback control process where the model is tuned using hyperparameter tuning unless the desired performance is obtained. The infection prediction error *e*_*inf*_obtained from the evaluation is fed back to tune the hyperparameters. The tuned model f*(*inf*) is deployed for predicting infections accurately. The deployed model is updated in real-time using the model feedback loop when the infection data is updated. The healthcare organizations and the government then use the deployed model to predict the infections. This is by providing the input arguments, country for which the prediction is required, and the duration of prediction *C*, *t*. The number of infections for the prediction period *Inf_t_* is sent to the healthcare organizations and the government.

### Dataset

**FIGURE 3:**
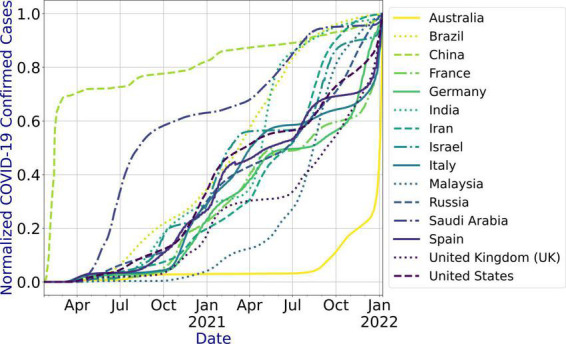
COVID-19 infections’ data trend for the countries under study.

To evaluate the performance of our proposed system, we developed the prediction models for fifteen countries based on the literature (Table 1). We used the Johns Hopkins COVID-19 dataset that is updated daily.^6^ Table 2 presents the countries for which the prediction models are developed, the features of the dataset, data update frequency, and the period for which the COVID-19 infections data are extracted for the countries under study. The dataset has no outliers and missing values. We used the number of confirmed cases for each country to develop the models. Figure 3 shows the infection trend for the considered countries. As shown in the figure, the distribution of the infection growth for each country is different. In this paper, we use country-level data for the evaluation as the dataset does not include city-level or state-level data for the countries under study. However, the system can be used for city-level or state-level infection data as well.

**TABLE 2:** Characteristics of the COVID-19 dataset used in the experiments.

Countries	Features	Update frequency	Considered period for the Covid-19 infections
Australia, Brazil, China, France, Germany, India, Iran, Israel, Italy, Malaysia, Russia, Saudi Arabia, Spain, United Kingdom, and United States	Province/state, country/region, last update, number of confirmed cases, number of recovered cases, and number of deaths	Daily	22/01/2020–08/01/2022

### Experiments and evaluation metrics

To predict the COVID-19 infections for the countries under study, we used our proposed system that selected the most accurate machine/deep learning model based on the temporal distribution of the infection evolution for a country (31) as stated in Figure 1. For each country under study, we compared the performance of the model selected using our proposed system with the outperforming model(s) in the literature for that country (Table 1). Table 3 presents the selected model and the models used for the comparison for each country. The description and the parameters for the models are listed in Table 4.

**TABLE 3:** Prediction models used for the countries under study.

Country	Infection’s trend	Automated AI selected model	Model(s) used for comparison
China	Exponential + linear	HLT	Bi-LSTM (25)
France			ARIMA (27)
Germany			Bi-LSTM (25)
Italy			ARIMA (24, 27) and Bi-LSTM (25)
Malaysia			ARIMA (28)
Australia	Polynomial	QT	LSTM (26)
Iran			Bi-GRU (26)
Russia			Bi-LSTM (25)
Spain			SutteARIMA (23), Bi-LSTM (25) and ARIMA (27)
UK			Bi-LSTM (25)
US	Linear	LT	ARIMA (24) and Bi-LSTM (25)
Israel			Bi-LSTM (25)
Brazil	Exponential + damping	DT	Bi-LSTM (25)
India			Bi-LSTM (25)
Saudi Arabia			ARIMA (29)

**TABLE 4:** Description and parameters of the prediction models used in the experiments.

Model	Description	Parameter
HLT	Allows forecasting of data with a trend. It is exponential smoothing applied to both the average value in the series (level) as well as the trend (47).	Smoothing parameters for level (α) and trend (β)
QT	Develops a polynomial relationship between time and the infection data (31).	Degree of polynomial
LT	Develops a linear relationship between time and the infection data (31). It is suitable for the time series where the local mean is increasing gradually over time at a constant rate.	Not applicable
DT	Extends the HLT model by adding a damping parameter that dampens the steep increasing forecast of HLT to a flat trend in the future (46).	Smoothing parameters for level (α), trend (β), and damping parameter (Φ)
LSTM	LSTM is a recurrent neural network that is capable of learning long-term dependencies. The main concepts of LSTM are the cell state and the gates. The cell state acts as a data transmission channel that transfers relative information to the chain of neural networks. Gates are the way to decide on what information to keep or forget based on the relevance during the training.	input size, number of neurons, epochs, activation function, and optimizer
Bi-LSTM	A recurrent neural network model consisting of two LSTM networks, one in forward direction (previous timestamp to future) and backward direction (future to previous timestamps).	
Bi-GRU	A neural network model consisting of two GRU networks, one taking input in forward direction and the other in backward direction. It is a bidirectional recurrent neural network consisting of input and forget gates. GRU are similar to LSTM cells but do not maintain an internal cell state	
ARIMA	Combines the autoregressive (AR) and the moving average (MA) models (29). AR develops a linear regression model with lagged infections as the independent variables and the MA develops a linear regression model using lagged prediction errors as the independent variables. A non-stationary time series data trend should be transformed into a stationary one, using differencing, to apply ARIMA.	Orders of lag observations (p), differencing (d), and moving average (q)
SutteARIMA	Averages alpha-Sutte and ARIMA prediction models (23). Alpha-Sutte is based on the moving average method and uses the infection’s data for the past 4 timestamps to predict infection for the next timestamp.	Orders of lag observations (p), differencing (d), and moving average (q)

To develop the prediction models, we create a separate dataset for each considered country. We use 70% of the dataset (i.e., 22/01/2020—06/06/2021) for training (develop) the model and 30% of the dataset (i.e., 07/06/2021–08/01/2022) for validating the developed model. We first developed a model for each country using the training dataset for that country. We then validated the developed model by predicting the number of infections for the validation period, i.e., 07/06/2021–08/01/2022, and comparing the predicted values with the actual ones. In addition, we developed the outperforming model(s) for each country under study based on the literature (Table 1) and predicted the infections using the developed model(s). We evaluate the performance of the models in terms of RMSE and MAPE that are computed using Equations (1) and (2), respectively.


(1) $$R\,M\,S\,E=\sqrt{\frac{\sum_{T=1}^{n}{\left(I\,n\,f\,e\,c\,t\,i\,o\,n\,s_{T}^{a\,c\,t\,u\,a\,l}-I\,n\,f\,e\,c\,t\,i\,o\,n\,s_{T}^{p\,r\,e\,d\,i\,c\,t\,e\,d}\right)}^{2}}{n}}$$

(2) $$MAPE=\left(\frac{1}{n}\sum_{T=1}^{n}\frac{\left|Infections_{T}^{actual}-Infections_{T}^{predicted}\right|}{Infections_{T}^{actual}}\right)\times 100\%$$

where n is the total number of days for which the infections are predicted

To tune the hyperparameters for the considered models, we implement each model with varying parameters’ values and select the values that result in the least MAPE. In particular, to obtain the values of α and β parameters for HLT model, we implement the model with varying values of the parameters between [0, 1] at an interval of 0.1, i.e., (α = 0, β = 0), (α = 0, β = 0.1), …, (α = 0.2, β = 0), (α = 0.2, β = 0.1),… (α = 1, β = 1). The combination of values that return the minimum MAPE is selected. For QT, we implement the model for varying degrees of polynomial between [1, 10] and selected the degree resulting in the least MAPE value. To obtain the values of α, β, Ø parameters for the DT model, we implement the model with varying values of the parameters between [0, 1] at an interval of 0.1 and selected the combination of values that return the minimum MAPE. To obtain the values of input size, number of neurons, epochs, activation function, and optimizer for LSTM, Bi-LSTM, and Bi-GRU models, we first determine the values of input size, number of neurons, and epochs by brute-force method while using Rectified Linear Unit (ReLU) activation function and Adaptive Movement Estimation (Adam) optimizer. We then vary the activation function and optimizer by keeping other parameters constant at their optimal values. The input sizes of 10, 50, 100, 200, and 250 are considered for the experiments. The different values used for epochs are 100, 200, 300, 400, and 500. However, for Italy, 1500 epochs are used as the model did not converge with 500 epochs. The number of neurons is varied from 100 to 1,000 at an interval of 100. The different activation functions used are ReLU, Softplus, Softmax, Softsign, Scaled Exponential Linear Unit (SELU), Linear, Hard_sigmoid, Sigmoid, Hyperbolic Tangent (Tanh), and Exponential Linear Unit (ELU). The optimizers used for tuning are Adam, Adadelta, Adaptive Gradient (AdaGrad), Adamax, Nesterov-accelerated Adaptive Moment Estimation (Nadam), Stochastic Gradient Descent (SGD), and Root Mean Square Propagation (RMSprop). The Mean Squared Error (MSE) loss function is used for LSTM, Bi-LSTM, and Bi-GRU models. To yield parameters’ values for the ARIMA and SutteARIMA models, we first check the stationarity of the infection data and determine the value of d. This is by performing the statistical augmented Dickey-Fuller (ADF) test (33, 46) that checks the null hypothesis that the data is non-stationary and returns a probability score (*p*-value). A *p*-value < 0.05 indicates that the time series is stationary. If the *p*-value ≥ 0.05 (non-stationary time series), then the time series is differenced and the ADF test is performed again. This is repeated until the time series becomes stationary. The value of d is then equal to the number of times the series is differenced. After determining the value of d, we plot the Autocorrelation Function (ACF) and Partial Autocorrelation Function (PACF) plots for the differenced time series to determine the values of q and p, respectively. The number of lags for which the ACF is outside the significant threshold represents the value of the parameter “q” value and the number of lags for which the PACF is outside the significant threshold represents the value of “p.”

## Results

### Hyperparameter tuning

**FIGURE 4:**
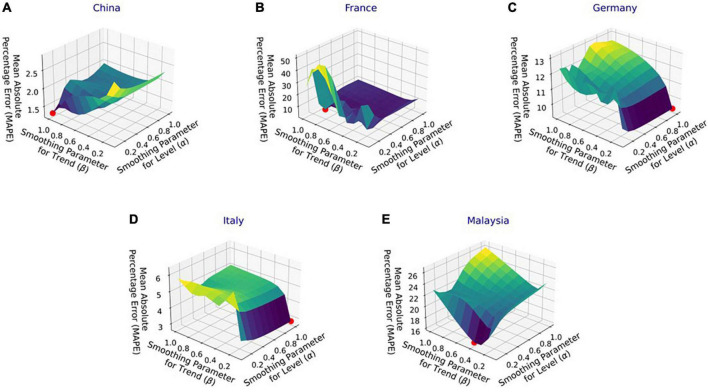
Performance of Holt’s linear trend (HLT) model with varying parameters’ values for the infection data in **(A)** China, **(B)** France, **(C)** Germany, **(D)** Italy, and **(E)** Malaysia.

**FIGURE 5:**
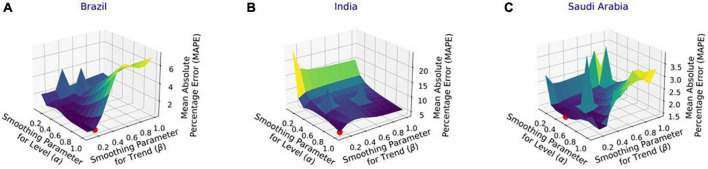
Performance of damped trend (DT) model with varying parameters’ values for the infection data in **(A)** Brazil, **(B)** India, and **(C)** Saudi Arabia.

**FIGURE 6:**
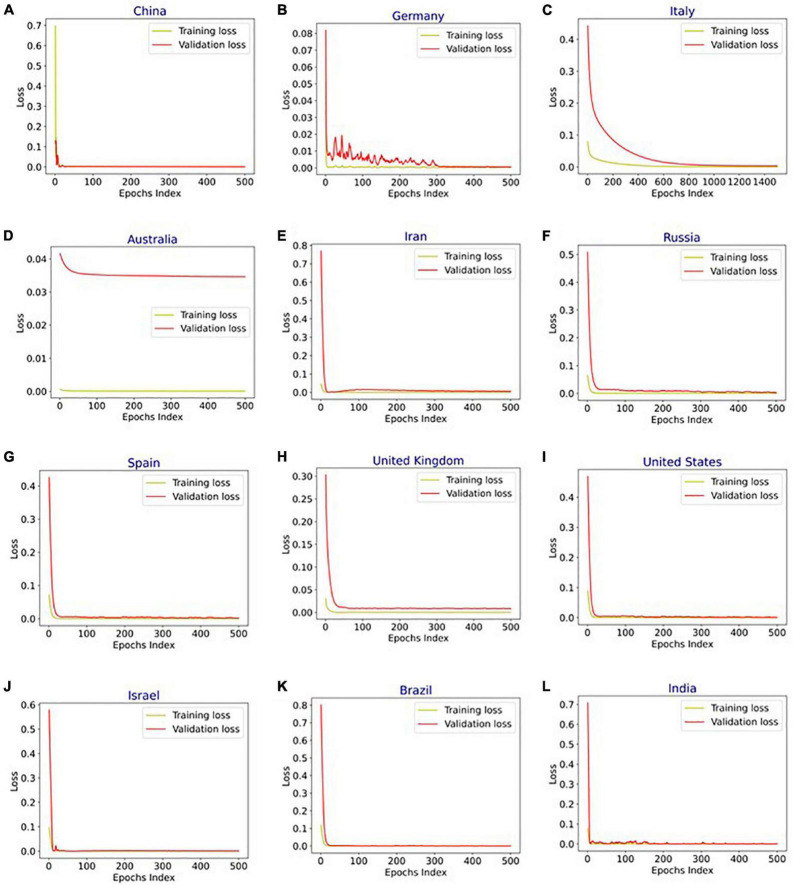
Training and validation loss vs. epochs for long short-term memory (LSTM), bidirectional-LSTM (Bi-LSTM), and bidirectional gated recurrent unit (Bi-GRU) models after hyperparameter tuning for infection data in **(A)** China (Bi-LSTM), **(B)** Germany (Bi-LSTM), **(C)** Italy (Bi-LSTM), **(D)** Australia (LSTM), **(E)** Iran (B-GRU), **(F)** Russia (Bi-LSTM), **(G)** Spain (Bi-LSTM), **(H)** United Kingdom (Bi-LSTM), **(I)** United States (Bi-LSTM), **(J)** Israel (Bi-LSTM), **(K)** Brazil (Bi-LSTM), and **(L)** India (Bi-LSTM).

**FIGURE 7:**
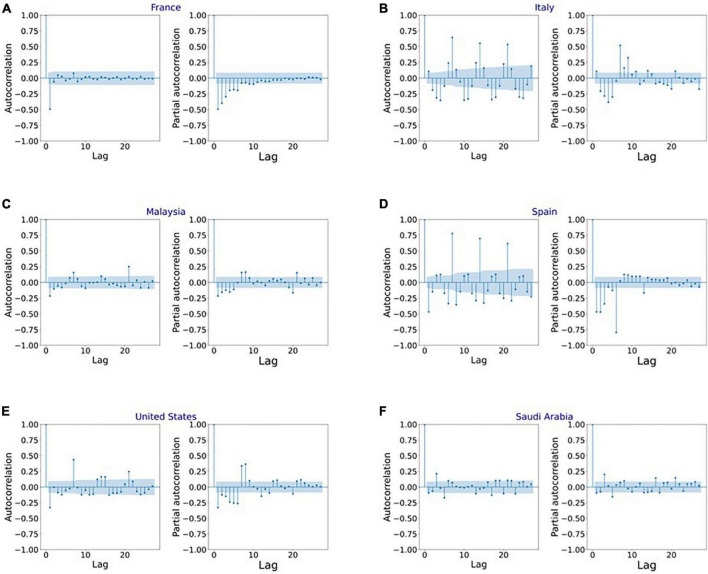
Autocorrelation function (ACF) and partial autocorrelation function (PACF) plots for the stationary infection data in **(A)** France, **(B)** Italy, **(C)** Malaysia, **(D)** Spain, **(E)** United States, and **(F)** Saudi Arabia.

Figure 4 shows the MAPE obtained by the HLT models, for different values of α and β, when developed for the infection data of China, France, Germany, Italy, and Malaysia. It shows that the minimum MAPE is obtained for (α, β) values of (0.1, 1.0), (0.3, 0.9), (1.0, 0.1), (1.0, 0.1), and (0.1, 0.4) for China, France, Germany, Italy, and Malaysia, respectively. We use these values to develop the prediction model for the corresponding countries. Figure 5 shows the MAPE obtained by the DT model, for different values of α and β, when developed for the infection data in Brazil, India, and Saudi Arabia. It shows that the minimum MAPE is obtained for (α, β) values of (1.0, 0.2), (1.0, 0.1), and (0.5, 0.1) for Brazil, India, and Saudi Arabia, respectively. We use these values to develop the prediction model. Figure 6 shows the training and validation losses over epochs for LSTM, Bi-LSTM, and Bi-GRU models for China, Germany, Italy, Australia, Iran, Russia, Spain, the United Kingdom, the United States, Israel, Brazil, and India. As shown in the figure, both training and validation losses converge, indicating a good fit. However, for Australia (Figure 6D), there is a gap between the training and validation losses indicating unrepresentative training dataset. This is because the number of infections for Australia increased rapidly during the validation period, as shown in Figure 3, which is not captured by the model develop using the training dataset. For the ARIMA model, we first perform the ADF test to check the stationarity of time series data for France, Italy, Malaysia, Spain, the United States, and Saudi Arabia. The *p*-values obtained for Malaysia, Spain, the United States, and Saudi Arabia after the second-order are 0.000000, 0.000000, 0.000092, and 0.000117, respectively. The *p*-values < 0.05 for these countries indicate that the time series becomes stationary after second-order differencing. Consequently, the value of d is set to 2 for these countries. For France and Italy, *p*-values < 0.05, i.e., 0.003894 and 0.048181, respectively, are obtained after first order differencing. However, the ACF plots for the first ordered differenced infection data of France and Italy do not converge to zero. Consequently, we differenced the time series for these countries one more time and select *d* = 2 for France and Italy after obtaining a *p*-value of 0.000000 and 0.001730, respectively. Figure 7 shows the ACF and PACF plots for the stationary infection data, i.e., after second-order differencing, for France, Italy, Malaysia, Spain, the United States, and Saudi Arabia. As depicted in Figure 7A, 1 lag value is outside the significant threshold in the ACF plot for France indicating *q* = 1. Moreover, 10 values in the PACF plot are outside the threshold indicating *p* = 10. Similarly, (p, q) values for Italy, Malaysia, Spain, the United States, and Saudi Arabia are (5, 7), (5, 2), (6, 8), (9, 1), and (3, 1) as shown in Figures 7B–F), respectively. Table 5 shows the optimal values of parameters for the developed models.

**TABLE 5:** Optimal values of parameters obtained after hyperparameter tuning for the models used in the experiments.

Model	Country	Optimal parameters’ values
HLT	China	α = 0.1, β = 1.0
	France	α = 0.3, β = 0.9
	Germany	α = 1.0, β = 0.1
	Italy	α = 1.0, β = 0.1
	Malaysia	α = 0.1, β = 0.4
QT	Australia	Degree = 5
	Iran	Degree = 2
	Russia	Degree = 2
	Spain	Degree = 3
	United Kingdom	Degree = 2
DT	Brazil	α = 1.0, β = 0.2, Φ = 0.99
	India	α = 1.0, β = 0.1, Φ = 0.99
	Saudi Arabia	α = 0.5, β = 0.1, Φ = 0.99
LSTM	Australia	Input size = 250, neurons = 100, epochs = 500, activation function = ReLU, optimizer = SGD
Bi-LSTM	China	Input size = 250, neurons = 100, epochs = 500, activation function = SELU, optimizer = Adamax
	Germany	Input size = 250, neurons = 100, epochs = 500, activation function = SELU, optimizer = Adadelta
	Italy	Input size = 250, neurons = 100, epochs = 1,500, activation function = ReLU, optimizer = SGD
	Russia, Spain, United States, and Brazil	Input size = 250, neurons = 100, epochs = 500, activation function = ReLU, optimizer = Adadelta
	United Kingdom	Input size = 250, neurons = 100, epochs = 500, activation function = Softsign, optimizer = Adadelta
	Israel	Input size = 250, neurons = 100, epochs = 500, activation function = ReLU, optimizer = Adam
	India	Input size = 250, neurons = 100, epochs = 500, activation function = ReLU, optimizer = Nadam
Bi-GRU	Iran	Input size = 250, neurons = 100, epochs = 500, activation function = ReLU, optimizer = Adam
ARIMA	France	*p* = 10, *q* = 2, *d* = 1
	Italy	*p* = 5, *q* = 2, *d* = 7
	Malaysia	*p* = 5, *q* = 2, *d* = 2
	Spain	*p* = 6, *q* = 2, *d* = 8
	United States	*p* = 9, *q* = 2, *d* = 1
	Saudi Arabia	*p* = 3, *q* = 2, *d* = 1

### COVID-19 predictions

**FIGURE 8:**
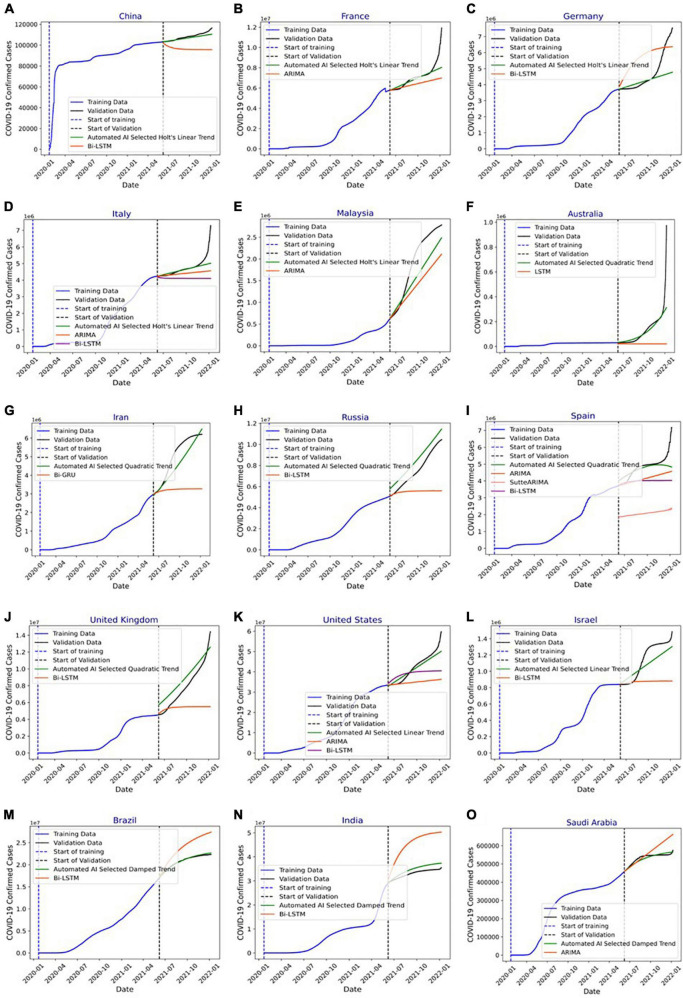
**(A)** Forecasting of COVID-19 infections in China using automated artificial intelligence-enabled system selected Holt’s linear trend (HLT) and state-of-the-art Bidirectional long short-term Memory (Bi-LSTM) models. **(B)** Forecasting of COVID-19 infections in France using Automated Artificial Intelligence-enabled system selected HLT and state-of-the-art Autoregressive Integrated Moving Average (ARIMA) models. **(C)** Forecasting of COVID-19 infections in Germany using Automated Artificial Intelligence-enabled system selected HLT and state-of-the-art Bi-LSTM models. **(D)** Forecasting of COVID-19 infections in Italy using Automated Artificial Intelligence-enabled system selected HLT and state-of-the-art ARIMA and Bi-LSTM models. **(E)** Forecasting of COVID-19 infections in Malaysia using Automated Artificial Intelligence-enabled system selected HLT and state-of-the-art ARIMA models. **(F)** Forecasting of COVID-19 infections in Australia using Automated Artificial Intelligence-enabled system selected QT and state-of-the-art LSTM models. **(G)** Forecasting of COVID-19 infections in Iran using Automated Artificial Intelligence-enabled system selected QT and state-of-the-art Bi-GRU models. **(H)** Forecasting of COVID-19 infections in Russia using Automated Artificial Intelligence- enabled system selected Quadratic Trend (QT) and state-of-the-art Bi-LSTM models. **(I)** Forecasting of COVID-19 infections in Spain using Automated Artificial Intelligence-enabled system selected QT and state-of-the-art ARIMA, SutteARIMA, and Bi-LSTM models. **(J)** Forecasting of COVID-19 infections in the United Kingdom using Automated Artificial Intelligence-enabled system selected QT and state-of-the-art Bi-LSTM models. **(K)** Forecasting of COVID-19 infections in the United States using Automated Artificial Intelligence-enabled system selected Linear Trend (LT) and state-of-the-art ARIMA and Bi-LSTM models. **(L)** Forecasting of COVID-19 infections in Israel using Automated Artificial Intelligence-enabled system selected LT and state-of-the-art Bi-LSTM models. **(M)** Forecasting of COVID-19 infections in Brazil using Automated Artificial Intelligence-enabled system selected Damped Trend (DT) and state-of-the-art Bi-LSTM models. **(N)** Forecasting of COVID-19 infections in India using Automated Artificial Intelligence-enabled system selected DT and state-of-the-art Bi-LSTM models, and **(O)** forecasting of COVID-19 infections in Saudi Arabia using Automated Artificial Intelligence-enabled system selected DT and state-of-the-art ARIMA models.

Figure 8A shows COVID-19 confirmed cases for the training and validation datasets for China. In addition, it indicates the number of infections forecasted by the HLT model selected using the proposed automated AI system and the Bi-LSTM model from the literature (25). It shows that HLT model predicts the infections with more accuracy compared to Bi-LSTM. This is because HLT fits well the exponential + linear infection trend for China. The (MAPE, RMSE) values using HLT and Bi-LSTM models for China are (1.29, 1934.36) and (11.39, 13331.86), respectively. Figure 8B shows the predicted infections for France using the proposed automated AI-selected HLT model and state-of-the-art ARIMA model (27). It shows that HLT outperforms ARIMA. As depicted in Figure 8B, HLT model predicts with lower error for the validation period where the infection’s trend is linear than where the trend is exponential. The prediction error for HLT increases as the infection grows exponentially toward the end of the validation period, which is not captured by the model. The (MAPE, RMSE) values using HLT and ARIMA models for France are (3.87, 702931.85) and (9.39, 1155417.17), respectively. The prediction for Germany using automated AI-selected HLT and state-of-the-art Bi-LSTM (25) is shown in Figure 8C. HLT outperforms Bi-LSTM as it can capture the exponential + linear infection trend for Germany. However, similar to Figure 8B, the prediction error by HLT for Germany (Figure 8C) increases when the validation infection data exhibits an exponential trend. The (MAPE, RMSE) values using HLT and Bi-LSTM models for Germany are (9.37, 967916.97) and (28.01, 1321353.74), respectively. Figure 8D shows COVID-19 prediction for Italy using automated AI-selected HLT and state-of-the-art ARIMA (24, 27) and Bi-LSTM (25) models. The HLT model outperforms ARIMA and Bi-LSTM models. The (MAPE, RMSE) values using HLT, ARIMA, and Bi-LSTM models for Italy are (2.84, 389747.98), (6.56, 581053.16), and (12.41, 837410.43), respectively. The prediction results for Malaysia using our automated AI-selected HLT model and state-of-the-art ARIMA model (28) are presented in Figure 8E. HLT captures the infection trend for Malaysia and outperforms ARIMA in predicting COVID-19 infections. The (MAPE, RMSE) values using HLT and ARIMA models for Malaysia are (16.37, 412523.95) and (23.23, 617834.31), respectively.

**FIGURE 9:**
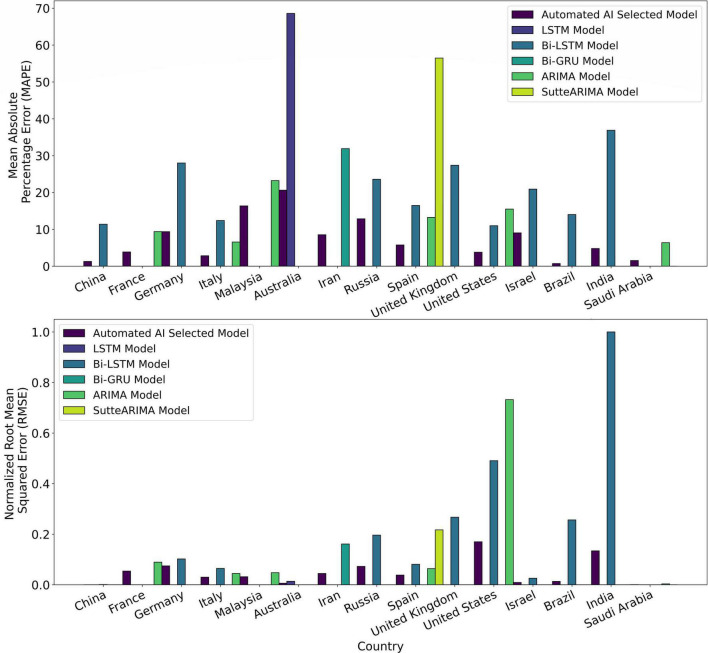
Mean absolute percentage error (MAPE) and normalized root mean squared error (RMSE) of the Automated Artificial Intelligence-enabled system selected and state-of-the-art models for the countries under study.

Figure 8F shows the prediction results for Australia using automated AI-selected QT and state-of-the-art LSTM (26). The (MAPE, RMSE) values using QT and LSTM models for Australia are (20.64, 80417.79), and (68.60, 181145.56), respectively. Figure 8G shows the prediction results for Iran using automated AI-selected QT and Bi-GRU (26). The (MAPE, RMSE) values using QT and Bi-GRU models for Iran are (8.54, 579794.14) and (31.90, 2086139.84), respectively. Figure 8H shows COVID-19 predictions for Russia using automated AI-selected QT and Bi-LSTM (25). It depicts that QT outperforms Bi-LSTM as it can capture the polynomial trend of the infection data in Russia. The (MAPE, RMSE) values using QT and Bi-LSTM models for Russia are (12.87, 941065.72) and (23.58, 2536117.98), respectively. Figure 8I shows the prediction results for Spain using automated AI-selected QT and state-of-the-art ARIMA (27), SutteARIMA (23), and Bi-LSTM (25). The (MAPE, RMSE) values using QT, ARIMA, SutteARIMA, and Bi-LSTM models for Spain are (5.77, 497155.75), (13.26, 825509.28), (56.48, 2804433.84), and (16.48, 1047913.19), respectively. Figure 8J shows the prediction results for the United Kingdom using automated AI-selected QT and Bi-LSTM (25). The (MAPE, RMSE) values using QT and Bi-LSTM models for the United Kingdom are (16.57, 1167306.58) and (27.40, 3450595.03), respectively. Figure 8K shows the COVID19 infection prediction for the United States using LT, ARIMA (24), and Bi-LSTM (25). The (MAPE, RMSE) values using LT, ARIMA, and Bi-LSTM models for the United States are (3.79, 2197376.04), (15.5, 9450564.22), and (10.99, 6337067.40) respectively. Figure 8L shows the prediction results for Israel using automated selected LT and Bi-LSTM (25). The (MAPE, RMSE) values using LT and Bi-LSTM models for Israel are (9.06, 119886.19) and (20.91, 335433.23), respectively. Figures 8M,N) show the prediction results for Brazil and India, respectively, using automated AI-selected DT models and Bi-LSTM models (25). They show that DT outperforms Bi-LSTM for both Brazil and India as it can accurately DT capture the exponential + damping trend of infection growth. The (MAPE, RMSE) values using DT and Bi-LSTM models for Brazil are (0.73, 175627.67) and (14.02, 3313775.77), respectively. The (MAPE, RMSE) values using DT and Bi-LSTM models for India are (4.79, 1732187.64) and (36.89, 12906730.59), respectively. Figure 8O shows the prediction results for Saudi Arabia using automated AI selected DT and ARIMA (29). The (MAPE, RMSE) values using DT and ARIMA models for Saudi Arabia are (1.54, 9909.39) and (6.37, 47768.10), respectively. Figure 9 show the MAPE and RMSE obtained by the model selected using the proposed system and state-of-the-art approaches for each country under study. It shows that the selected models outperform the approaches in the literature for each country. In summary, the proposed system predicts COVID-19 infections with an average MAPE and RMSE of 7.87 and 665052.14, respectively. The average MAPE values for state-of-the-art Bi-LSTM, ARIMA, LSTM, Bi-GRU, and SutteARIMA models are 20.21, 12.38, 68.60, 31.90, and 56.48, respectively, whereas the average RMSE values are 3209972.92, 2113024.38, 181145.57, 2086139.84, and 2804433.85, respectively.

## Discussion

Time series prediction is a useful method to predict the dynamics of future infection data by using the influence of the trends, seasonality, and randomness of the historical data (31). Different machine learning algorithms have been used to analyze the data of epidemic and pandemic diseases such as influenzas A (H1N1), B, measles childhood disease (32), SARS, MERS, and COVID-19 outbreaks, at the country, regional or global level (31). Though any machine learning algorithm can produce reliable results at some level, time series algorithms are the most accurate approaches to studying epidemic and pandemic diseases because of their dynamic and temporal nature (33). Several machine learning and deep learning time series algorithms have been used in the literature to predict COVID-19 infections (23–29). The dominant concern in predicting infections for a country is the prediction’s accuracy, optimal resource management, and effective development of strategies. Our main goals are to (1) decide on an accurate time series learning algorithm for predictions, and (2) hyperparameter tuning for the selected algorithm. These algorithms are data-driven and are only suitable for a particular trend of the infection’s growth. Consequently, a single algorithm cannot be applied to predict infections’ spread in different countries. For instance, Autoregressive Integrated Moving Average (ARIMA) (29) cannot be used for prediction when the trend of infection’s growth linearizes/dampens over time. Furthermore, Holt’s Linear Trend (HLT) (47) model gives inaccurate prediction results if there exists a seasonality behavior in the infection’s growth. Table 6 presents the limitations of the models used in the literature (Table 1). In summary, Table 6 shows that no single algorithm can be used to accurately predict infections for all the countries in the world. This is because the infection trend is different from one country to another. Our proposed automated AI-enabled proactive preparedness real-time system analyzes a country’s infection trend and selects a time-series model which captures that particular trend. Our numerical experiments and comparative analysis show that the proposed system outperforms the state-of-the-art approaches for COVID-19 prediction. In particular, the proposed system predicts the number of infections with 68.60, 58.79, 69.90, 73.21, and 89.78% less MAPE, and 65.8150.18, 55.60, 72.20, and 82.27% lower RMSE than Bi-LSTM, ARIMA, LSTM, Bi-GRU, and SutteARIMA used in the literature, respectively.

**TABLE 6:** Limitations of time series algorithms.

Algorithm	Limitation
Autoregressive Integrated Moving Average (ARIMA)	Not suitable for infection’s trend that becomes linear or dampens over time
SutteARIMA	Not suitable for infection’s trend that increases exponentially
Holt’s linear trend	Not suitable for infection’s trend with seasonality
Trigonometric Exponential smoothing state-space model with Box-Cox transformation	Not suitable for infection’s trend that increases exponentially
Cubic smoothing spline	Not suitable for infection’s trend having a high difference in the number of infections between consecutive time intervals
Support vector regression	Not suitable for infection’s trend with randomness
Long short-term memory (LSTM), Bi-LSTM, gated recurrent unit (GRU), and Bi-GRU	Time consuming, memory-intensive and the performance is sensitive to the initial values of hyperparameters
Autoregressive and Autoregressive Moving Average	Not suitable for infection’s trend whose average varies over time
Moving average	Can only predict a consistent change in infections over time

## Conclusion

Considering the dynamicity in the temporal distribution of infections over time among different countries, a single machine learning infection prediction algorithm cannot solely yield high accuracy for all the countries, and hence different models should be adopted for predicting infections in different countries. The selection of the model for a country is the main challenge as evaluating the performance of all the algorithms for a country and then selecting the most accurate model is a complex and inefficient process. For selecting the most accurate model the trend of the infection’s evolution for a country should be taken into consideration. Incorporating all these factors, a novel automated artificial intelligence-enabled proactive preparedness real-time system for accurate prediction of COVID-19 infection is proposed. We present the design, development, and implementation of the system. The proposed system selects the most accurate model based on the infection trend for a country, whereas the models in the literature are selected based on the popularity of the model or based on the performance of a models when used for other countries. The developed system performs efficiently, with an average reduction of 72.1% in MAPE and 65.2% in RMSE compared to state-of-the-art approaches. Consequently, the system will aid governments to tailor the precautionary measures in place to tackle a pandemic, such as COVID-19, and develop an effective plan to manage the medical resources efficiently. For future research work, a large spectrum of countries will be considered to evaluate the proposed system. In addition, efficient methods for models’ calibrations will be investigated.

## Data availability statement

We used publicly available Johns Hopkins COVID-19 dataset in this study. This data is updated daily and can be found here: https://data.humdata.org/dataset/novel-coronavirus-2019-ncov-cases.

## Author contributions

LI conceived the topic, conducted the conceptualization, design, investigation, methodology, experiments, prepared and wrote the first draft of the manuscript, and manuscript review and editing. HM participated in the investigation, experiments, and first draft preparation. YA participated in writing the security part. FF contributed to the design, methodology, and manuscript review and editing. GK contributed to the introduction and manuscript review and editing. SRBA contributed to the manuscript review and editing. All authors contributed to the manuscript revision, read, and approved the submitted version.

## Footnotes

1 https://www.who.int/emergencies/diseases/novel-coronavirus-2019 (last accessed on May 23, 2022).

2 https://covid19.who.int/ (last accessed on May 23, 2022).

3 https://www.cdc.gov/flu/pandemic-resources/2009-h1n1-pandemic.html (last accessed on May 16, 2022).

4 https://www.cdc.gov/flu/pandemic-resources/pandemic-timeline-1930-and-beyond.htm (last accessed on May 16, 2022).

5 https://apps.nsa.gov/iaarchive/programs/iad-initiatives/cnsa-suite.cfm (last accessed on May 16, 2022).

6 https://data.humdata.org/dataset/novel-coronavirus-2019-ncov-cases (last accessed on May 16, 2022).
